# Sleep-Wake Rhythm and Oscillatory Pattern Analysis in a Multiple Hit Schizophrenia Rat Model (Wisket)

**DOI:** 10.3389/fnbeh.2021.799271

**Published:** 2022-01-28

**Authors:** Leatitia Gabriella Adlan, Mátyás Csordás-Nagy, Balázs Bodosi, György Kalmár, László G. Nyúl, Attila Nagy, Gabriella Kekesi, Alexandra Büki, Gyongyi Horvath

**Affiliations:** ^1^Department of Physiology, Albert Szent-Györgyi Medical School, University of Szeged, Szeged, Hungary; ^2^Department of Technical Informatics, Faculty of Science and Informatics, Institute of Informatics, University of Szeged, Szeged, Hungary; ^3^Department of Image Processing and Computer Graphics, Faculty of Science and Informatics, Institute of Informatics, University of Szeged, Szeged, Hungary

**Keywords:** oscillatory activity, EEG, schizophrenia, circadian rhythm, multiple hit model

## Abstract

Electroencephalography studies in schizophrenia reported impairments in circadian rhythm and oscillatory activity, which may reflect the deficits in cognitive and sensory processing. The current study evaluated the circadian rhythm and the state-dependent oscillatory pattern in control Wistar and a multiple hit schizophrenia rat model (Wisket) using custom-made software for identification of the artifacts and the classification of sleep-wake stages and the active and quiet awake substages. The Wisket animals have a clear light-dark cycle similar to controls, and their sleep-wake rhythm showed only a tendency to spend more time in non-rapid eye movement (NREM) and less in rapid eye movement (REM) stages. In spite of the weak diurnal variation in oscillation in both groups, the Wisket rats had higher power in the low-frequency delta, alpha, and beta bands and lower power in the high-frequency theta and gamma bands in most stages. Furthermore, the significant differences between the two groups were pronounced in the active waking substage. These data suggest that the special changes in the oscillatory pattern of this schizophrenia rat model may have a significant role in the impaired cognitive functions observed in previous studies.

## Introduction

Schizophrenia is a chronic and multidimensional neuropsychiatric disorder with devastating consequences for patient outcomes. Although not included in its diagnostic criteria, sleep disturbances are consistently reported in patients, including disrupted circadian rhythms of activity, sleep-wake, and oscillatory patterns obtained by electroencephalography (EEG) (Felder et al., 1994; Sponheim et al., 1994, 2003; Omori et al., 1995; Wirz-Justice et al., 2001; Symond et al., 2005; Rockstroh et al., 2007; Bob et al., 2008; Boutros et al., 2008; Afonso et al., 2011; Pritchett et al., 2012; Başar, 2013; Lakatos et al., 2013; Uhlhaas and Singer, 2013; Featherstone et al., 2015; Tam et al., 2015; Manoach et al., 2016; Newson and Thiagarajan, 2019). All these abnormalities may be implicated in impaired sleep-dependent memory consolidation and may represent an endophenotype for schizophrenia that contributes to abnormal cognitive and sensory performances (Manoach et al., 2016). Unfortunately, the results are very controversial and they may depend on the subtypes or phases of this disease or type of drug treatment. Furthermore, the EEG abnormalities are neither consistent nor unique in schizophrenia as most of these signs are also reported in other mental disorders such as major depression and autism spectrum disorder (Cohrs, 2008; O’Reilly et al., 2017).

Rodent models are essential for understanding brain function in health and disease. Several animal models of schizophrenia have abnormalities in their circadian rhythm and oscillatory pattern, but most of these results obtained in mutant mice with modification of only one gene transcription, e.g., disrupted-in-schizophrenia 1 (DISC1) gene, metabotropic glutamate 5 receptors (mGLUR5), voltage gated L-type calcium channels, serine racemase enzyme (responsible for N-methyl-D-aspartate (NMDA) receptor function), GluA1 subunit of α-amino-3-hydroxy-5-methyl-4-izoxazolepropionic acid (AMPA) receptors, or stable tubule only polypeptide (STOP) (Ahnaou et al., 2015b; Kumar et al., 2015; Profitt et al., 2016; Ang et al., 2018; Aguilar et al., 2020). Only few studies investigated single hit rat models (Ahnaou et al., 2007; Phillips et al., 2012a; Valdés-Cruz et al., 2012). Most of these animal models possess few disturbances in their sleep-wake rhythm and/or oscillatory pattern, with some contradictions between the studies.

Since the etiology of schizophrenia involves the interaction among genetic, developmental, and environmental factors, multiple hit translational models might provide animals with a high level of constructive and face validities with a wider range of schizophrenia-related signs. Therefore, a triple hit rat model, named Wisket, was developed in our laboratory by combining developmental (postweaning social isolation for 4 weeks), pharmacological (NMDA receptor antagonist, ketamine, and treatment intraperitoneally: 30 mg/kg, 4 ml/kg body weight, daily, 5 times/week, 15 injections in total), and genetic (selective breeding based on behavioral phenotype for more than 30 generations) manipulations, as described previously (Supplementary Figure 1; Horvath et al., 2017, 2021a). Animals with decreased pain sensitivity, impaired sensory gating, and cognitive functions were selected for breeding. A wide range of disturbances was observed in Wisket animals, including impaired pain sensitivity, sensory gating, cognition and alterations in opioid, cannabinoid, oxytocin, and dopamine receptors’ (D_2_R and D_1_R) signaling and/or expression (Petrovszki et al., 2013; Szûcs et al., 2016b,a, 2020; Horvath et al., 2017, 2021b; Banki et al., 2020). Furthermore, the Wisket model rats exhibited altered daily rhythm in the gross motor activity and body temperature investigated by telemetry, and changes in oscillatory patterns and evoked potentials detected in a short-term EEG paradigm (Horvath et al., 2015, 2016). To characterize further the possible disturbances in these animals, the aim of this study was to determine their sleep-wake rhythm and oscillatory pattern with the application of EEG in freely moving conditions.

Traditionally, the classification of the wake-sleep stages in the EEG records is performed by trained human experts *via* visual inspection of the EEG features, which is a laborious task prone to interindividual variability and it also requires considerable time and specialized knowledge about sleep in rodents. Considerable progress in computational technologies has made possible an improvement in automated sleep scoring algorithms (Miladinović et al., 2019; Yamabe et al., 2019). Therefore, the aim of this study was to characterize the sleep/wake states architecture and sleep/wake-related oscillations in the Wisket rat model of schizophrenia by more accurate software.

## Materials and Methods

### Animals

Male, adult Wistar (control) and Wisket rats (4–6 months old) were involved in the study. All experiments were carried out with the approval of the Hungarian Ethical Committee for Animal Research (registration number: XIV/1248/2018) and in accordance with the guidelines set by the Government of Hungary and EU Directive 2010/63EU for animal experiments. The animals were kept with a 12 h light/dark cycle under conditions of controlled temperature (23 ± 1°C) and humidity (55 ± 10%).

### Surgical Procedure

The whole setup of the surgery was similar as described previously (Bodosi et al., 2000, 2004; Horvath et al., 2016). Briefly, the rats were anesthetized with a mixture of ketamine hydrochloride (Calypsol, Gedeon Richter Plc., Budapest, Hungary; 72 mg/kg) and xylazine (CP-Xylazin, Produlab Pharma B.V. Raamsdonksveer, Netherlands, 8 mg/kg) administered intraperitoneally, and transferred into a stereotaxic frame. For EEG recording, each animal was implanted with stainless steel screws over the parietal cortex (from bregma: −2.3 and 2.4 mm right to the midline), occipital cortex (ground electrode: −6.1 and 2.4 mm right to the midline), and the cerebellum (reference: −10.5 and 0.5 mm right to the midline). A thermistor was placed over the left parietal cortex to record brain cortical temperature (Tc). Finally, the electrodes were connected with enamel-coated copper wires to a miniature connector, which was fixed to the skull with dental cement.

### Experimental Paradigm

After the surgery, the rats were housed individually in custom-made clear plexiglass cages (25 cm × 28 cm × 50 cm), to avoid the removal of the implanted devices by their cage mate. The rats were in visual, auditory, and olfactory contact with other rats to minimalize the effect of isolation rearing (Hurst et al., 1999). They were allowed to recover for 1 week with access to water and food *ad libitum*. The room was sound attenuated. During this period, the rats were connected to the recording tether and habituated to the experimental conditions. After the recovery period, EEG was recorded for 23 h (1 h was devoted to taking care of the animals). However, due to technical reasons (the animals frequently disrupted the recording cable close to the light phase), 21 h-long EEG recordings were analyzed, including the total length of the light phase (12 h), and 9 h of the dark phase.

Two series of experiments were performed. In the first (matched) series, four rats (representing both control and Wisket animals; *n* = 8/group) were recorded simultaneously in each recording session. Furthermore, to determine whether the changes obtained between the two groups in the first series can be reproduced independently of the environmental circumstances, the second series of experiments was performed with a different group of animals, where the animals were unmatched (5 controls and 6 Wiskets, investigated on different days).

### Data Recording, Correction, and Classification

Cables from the electrodes were connected to a miniature microcontroller-based transmitter unit powered by a rechargeable Li-ion battery and tied to the cables. The housing of the cables was attached to a plastic bearing above the cage providing free rotation. Signals were amplified (2000×) and filtered (0.5−48 Hz), and were digitized by an inbuilt analog-to-digital converter at a sampling rate of 128 Hz. The resolution was 12 bits. The motor activity of the rats was detected by a 3 − axis accelerometer (type: LIS3LV02DQ; range: 0 − 6 G) within the unit, as described earlier (Bodosi et al., 2000, 2004). For scoring, the signals were transmitted at 2.4 GHz to a receiver unit attached to the PC over a USB connection. These units, including all PC and microcontroller programs, were developed and produced by one of the authors (BB). Data were analyzed in an 8-s epoch offline.

The EEG recordings contained artifacts, caused by short contact losses of the electrodes due to animal movements. During these periods, the EEG signal clipped and these sections’ spectra would mislead the spectrum-analysis approach presented in the article. To correct these sections, the EEG signals were preprocessed. First, the clipping signal parts were detected, and then they were analyzed and substituted. The replacement signal had the same length as the faulty section and was generated as a well-formed (colored) white Gaussian noise, which noise’s spectrum was formed to match the spectrum of the EEG signal surrounding the faulty section. Thus, the replacement does not affect the aggregated spectrum-based features extracted at the later stages of the analysis. The preprocessing method corrected 2% of the EEG dataset.

The next task was to classify the EEG signal into three wake-sleep stages: awake, non-rapid eye movement (NREM), and rapid eye movement (REM) stages. To achieve this automated classification, a machine-learning model was trained to predict the class label for 8 s-long EEG signal segments. To train such a model, we had access to a dataset containing 36 manually classified recorded days and approximately 10, 000 hand-labeled segments for each day. Our approach was to extract handcrafted features from the segments and use only these to predict a label. The generated features are the following: mean amplitude and SD values for the different frequency bands, the SD of the motion data, and a Petrosian Fractal Dimension value (Petrosian, 1995) of the EEG data. Then, the states of vigilance were determined over 8 s epochs as NREM sleep (high-amplitude slow waves, low level of body movements); REM sleep (highly regular theta activity in the EEG, low level of body movements with occasional twitches); and wakefulness (less regular theta activity, frequent body movements). Established in earlier studies, the awake stage was subdivided into two substages: active and quiet/inactive awake (Vyazovskiy and Tobler, 2005; Mofleh and Kocsis, 2021). The basis of the subdivision was the motor activity: longer than 1 s movement duration during the 8 s period referred to active substage. Based on these indicators, we trained a random forest classifier (Breiman, 2001), which achieved 92.8% accuracy measured with 10-fold cross-validation. This automated classifier method was used to predict labels for the EEG data served as the basis of the analysis results presented later in this article.

After the categorization of the states of vigilance, power spectra were computed by Fast Fourier Transform (FFT) for each stage and substages under a condition of 0.125°Hz resolution with a Hanning window. Total absolute power was calculated as the sum of squares of all frequency values contained in a given band. Relative band powers were expressed as power ratios of each frequency band to the total (z-score) in each 8 s bin. The resulting power spectra were divided and analyzed in different frequency ranges: delta (0.5−4 Hz), theta (4−8 Hz), alpha (8 − 12 Hz), beta (12 − 30 Hz), and low gamma (30–48 Hz) bands.

### Data and Statistical Analyses

The parameters were quantified and analyzed as means of values per hour for the 21 h (daily) and separately for the light and dark phases (diurnal). The mean duration per hour and the number and duration of episodes in different stages were determined. Regarding the detailed analysis of the oscillatory pattern, our preliminary data analysis revealed a separate or even opposite tendency in relative powers in narrow frequency ranges. Therefore, based on earlier results, we performed the statistical analysis for all of the data within a frequency band (delta, theta, alpha, beta, and gamma) to reveal fine-grained alterations between the two groups (Saravanapandian et al., 2020).

Data are expressed as means ± SEM. The parameters were compared with repeated and/or factorial ANOVA. When the global test was significant, the Fisher least significant difference (LSD) *post-hoc* test was used for the evaluation of the effects of various parameters. Statistical analysis was performed with STATISTICA 13.5.0.17 (TIBCO Software Inc., United States). Differences were considered significant for *p* < 0.05.

## Results

### Sleep-Wake Rhythm

Factorial ANOVA of the time spent in the different stages (awake, NREM, and REM) during the whole investigated period showed significant effects of the stage [*F*_(2_,_42)_ = 282.62; *p* < 0.001; Table 1]. Thus, both groups of animals spent a significantly shorter time in the REM stage compared to awake or NREM stages. The separate analysis of the active and quiet substages revealed significant effects of substage [*F*_(1_,_28)_ = 29.82; *p* < 0.001], thus, both groups of animals spent less time in quiet awake than in active awake substage (Table 1). Regarding the light:dark (diurnal) rhythm (phases), the ANOVA showed significant effects of the stage [*F*_(2_,_42)_ = 268.94; *p* < 0.001) and phase and stage interaction [*F*_(2_,_42)_ = 81.49; *p* < 0.001), thus, the animals in both groups spent more time in awake and less in NREM and REM stages of sleep during the dark phase compared to the light condition without significant differences between the two groups (Table 1). Furthermore, the separate analysis of the diurnal rhythm of the active and quiet substages revealed significant effects of substage [*F*_(1_,_28)_ = 29.92; *p* < 0.001], phase [*F*_(1_,_28)_ = 48.87; *p* < 0.001], and substage and phase interaction [*F*_(1_,_28)_ = 13.05; *p* < 0.005], thus both groups of animals spent a long time in both types of awake substages during the dark than in the light phase (Table 1).

**TABLE 1:** The mean per hour° ± °SEM duration of different stages and substages, the number and length of episodes in control and Wisket animals for the whole period and by light and dark phases.

STAGES/substages	Group	Duration (min)		No. of episodes		Length of episodes (s)		Total power	
**AWAKE**	Control	26.5 ± 0.89		25.9 ± 1.54		89.7 ± 11.17		0.021 ± 0.0017	
	Wisket	25.4 ± 1.01		23.9 ± 1.35		107.7 ± 22.72		0.025 ± 0.0019	
**Active**	Control	17.8 ± 1.76						0.022 ± 0.0018	
	Wisket	17.3 ± 1.96						0.025 ± 0.0020	
**Quiet**	Control	8.7 ± 1.46						0.019 ± 0.0017	
	Wisket	8.1 ± 1.47						0.023 ± 0.0017	
**NREM**	Control	26.4 ± 1.46		25.5 ± 1.65		64.0 ± 5.46		0.041 ± 0.0020	
	Wisket	28.2 ± 0.73		23.3 ± 1.42		73.9 ± 3.58		0.044 ± 0.0043	
**REM**	Control	**7.2 ± 0.94**		20.2 ± 3.77		21.8 ± 1.49		0.021 ± 0.0018	
	Wisket	**6.4 ± 0.46**		17.2 ± 1.86		22.5 ± 2.57		0.026 ± 0.0024	

		***Light***	***Dark***	***Light***	***Dark***	***Light***	***Dark***	***Light***	***Dark***

**AWAKE**	Control	20.2 ± 0.94	*34.8* ± *2.04*	27.5 ± 1.56	***23.7* ± *1.90***	60.5 ± 14.02	128.7 ± 16.08	0.021 ± 0.0017	0.021 ± 0.0017
	Wisket	17.1 ± 1.90	*36.6* ± *2.57*	25.6 ± 1.43	***21.5* ± *1.57***	49.2 ± 10.00	185.7 ± 22.85	0.024 ± 0.0021	0.025 ± 0.0018
**Active**	Control	12.9 ± 1.58	*24.3* ± *2.61*					0.022 ± 0.0019	0.021 ± 0.0017
	Wisket	11.1 ± 1.54	*25.6* ± *3.11*					0.025 ± 0.0022	0.026 ± 0.0019
**Quiet**	Control	7.3 ± 1.08	*10.5* ± *2.12*					0.019 ± 0.0014	0.019 ± 0.0015
	Wisket	5.9 ± 1.00	*11.0* ± *3.10*					0.023 ± 0.0017	0.023 ± 0.0018
**NREM**	Control	31.1 ± 1.20	*20.1* ± *2.09*	28.4 ± 2.09	*21.7* ± *1.38*	69.4 ± 5.31	56.7 ± 6.05	0.040 ± 0.0021	0.042 ± 0.0022
	Wisket	34.4 ± 1.29	*19.9* ± *1.94*	26.2 ± 1.17	*19.5* ± *1.92*	81.7 ± 4.85	63.4 ± 4.34	0.044 ± 0.0044	0.046 ± 0.0043
**REM**	Control	8.7 ± 1.29	*5.1* ± *0.75*	22.7 ± 4.41	*16.8* ± *3.05*	24.6 ± 2.12	**18.1 ± 1.74**	0.021 ± 0.0020	0.021 ± 0.0017
	Wisket	8.5 ± 0.76	*3.5* ± *0.72*	21.0 ± 2.02	*12.0* ± *2.06*	26.2 ± 3.56	**16.9 ± 1.51**	0.027 ± 0.0025	0.025 ± 0.0022

*Bolded data mean significant differences compared to NREM and awake stages. Data in italic letters show significant differences compared to the light phase. The underlined data show significant differences compared to active substages.*

The number and the length of episodes did not differ significantly between the two groups analyzed for the whole investigated period (Table 1). Regarding the diurnal rhythm in the number of episodes in awake, NREM, and REM stages, the ANOVA showed significant effects of the stage [*F*_(2_,_42)_ = 5.81; *p* < 0.01] and phase [*F*_(1_,_42)_ = 91.71; *p* < 0.001], thus, both groups of animals had fewer number in bouts of all stages during the dark phase, with a moderate tendency of decreased numbers in Wisket animals (Table 1). The analysis of the mean length of the episodes also showed significant effects of the stage [*F*_(2_,_42)_ = 24.75; *p* < 0.001], phase [*F*_(1_,_42)_ = 7.18; *p* < 0.05], and the stage and phase interaction [*F*_(2_,_42)_ = 15.01; *p* < 0.001], thus, both groups of animals had shorter REM sleep episodes compared to awake and NREM stages, and longer awake bouts during the dark phase (Table 1).

Regarding the unmatched groups, the Wisket animals, similarly to the matched series, did not show significant changes in these parameters compared to the control ones (data are not shown).

### Oscillatory Pattern

The oscillatory pattern of the EEG was analyzed next. Since no diurnal changes were obtained in the oscillations, power spectra were analyzed for the whole session independently of stages, and then for the three sleep-wake stages (REM, NREM, and awake) and the active and quiet awake substages, separately. To demonstrate the fine-grained distribution of the relative power, the ANOVA analysis was performed for all the data obtained within the different frequency bands (delta, theta, alpha, beta, and gamma).

Regarding the analysis of total absolute power, only a tendency of enhanced power was obtained in the Wisket animals by stages, substages, and the light-dark phases (Table 1).

**FIGURE 1:**
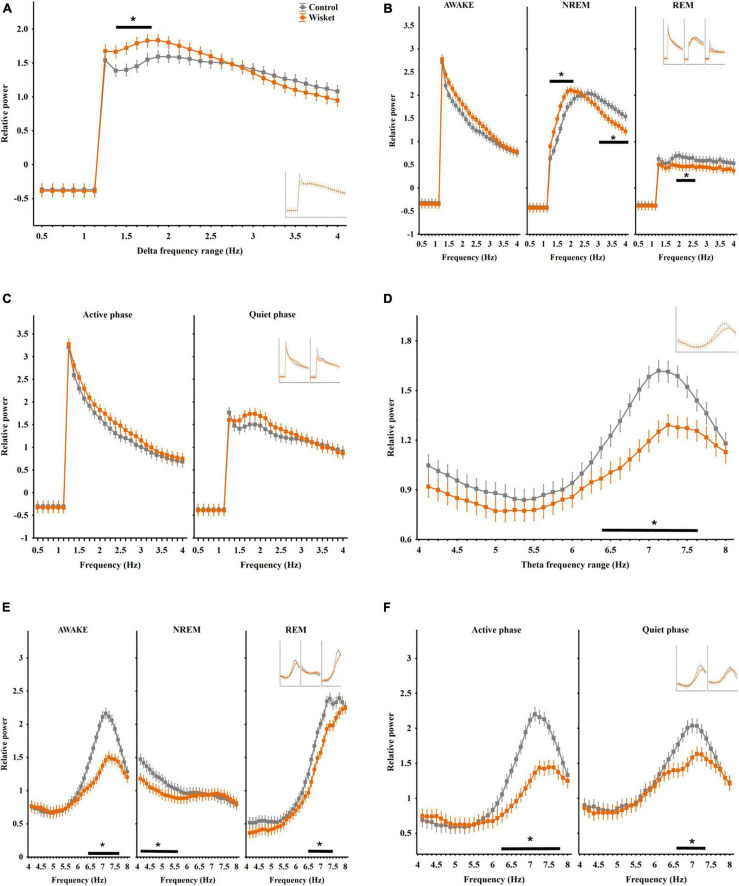
Relative EEG power differences between the two groups at the delta and theta frequency bands. **(A)** Total relative delta power (0.5–4 Hz). **(B)** Delta power in different stages. **(C)** Delta power in active and quiet awake substages. **(D)** Total relative theta power (4–8 Hz). **(E)** Theta power in different stages. **(F)** Theta power in active and quiet awake substages. Curves inserted in reduced size show the results obtained from unmatched control and Wisket animals. The symbol * shows QQsignificant (*p*° < °0.05) differences between the two groups.

Regarding the entire relative power irrespective of the stages in the delta band (0.5 − 4 Hz), factorial ANOVA showed significant effects of frequency [*F*_(28_,_406)_ = 157.82; *p* < 0.001] and group and frequency interaction [*F*_(28_,_406)_ = 1.52; *p* < 0.05]. The *post hoc* analyses revealed that the Wisket animals had significantly higher total relative power compared to control ones in the interval of 1.38 − 1.75 Hz (Figure 1A).

The analysis of the delta power by stages revealed significant effects of frequency [*F*_(28_,_1218)_ = 337.79; *p* < 0.001], stage [*F*_(2_,_1218)_ = 1638.03; *p* < 0.001], group and frequency interaction [*F*_(28_,_4065)_ = 2.41; *p* < 0.001], group and stage interaction [*F*_(2_,_1218)_ = 25.60; *p* < 0.001], frequency and stage interaction [*F*_(56_,_1218)_ = 46.25; *p* < 0.001], and group, frequency, and stage interaction [*F*_(56_,_1218)_ = 1.48; *p* < 0.05]. The *post hoc* analysis disclosed that the delta power was the lowest during the REM stage in both groups, with significantly lower in its middle range (1.88 − 2.5 Hz) in the model rats compared to control animals. Furthermore, the pattern of the delta spectrum differed between the two groups in the NREM stage, i.e., the Wisket animals had higher power at the lower frequency range (1.25 − 2 Hz), while they had significantly lower power at the higher frequency range (3 − 4 Hz) compared to control rats (Figure 1B).

The separate analysis of the delta band by substages revealed significant effects of group [*F*_(1_,_812)_ = 16.75; *p* < 0.001], frequency [*F*_(28_,_812)_ = 195.11; *p* < 0.001], substage [*F*_(1_,_812)_ = 40.67; *p* < 0.001)], and frequency and substage interaction [*F*_(28_,_812)_ = 13.11; *p* < 0.001]. Thus, the Wisket animals had a trend for higher delta power in both awake substages (Figure 1C).

Regarding the total relative power irrespective of the stages in the theta band (4 − 8°Hz), factorial ANOVA showed significant effects of group [*F*_(1_,_448)_ = 10.58; *p* < 0.001] and frequency [*F*_(31_,_448)_ = 25.05; *p* < 0.001]. The *post hoc* analysis showed that the Wisket animals had significantly lower power compared to control ones at the higher frequency theta band (6.38 − 7.62 Hz interval; Figure 1D).

The analysis of the theta power by stages revealed significant effects of group [*F*_(1_,_1344)_ = 206.48; *p* < 0.001], frequency [*F*_(31_,_1344)_ = 113.34; *p* < 0.001], stage [*F*_(2_,_1344)_ = 51.38; *p* < 0.001], group and frequency interaction [*F*_(31_,_1344)_ = 2.64; *p* < 0.001], group and stage interaction [*F*_(2_,_1344)_ = 10.92; *p* < 0.001], frequency and stage interaction [*F*_(62_,_1344)_ = 55.54; *p* < 0.001] and group, frequency, and stage interaction [*F*_(62_,_1344)_ = 1.48; *p* < 0.001]. Thus, the pattern of the theta spectrum differed between the stages with the lowest rise of the curve during NREM (Figure 1E). Furthermore, during NREM sleep the theta power at the lower frequency band (4.12 − 5.62 Hz) was significantly lower, while during awake and REM stages the higher frequency bands (6.38 − 7.62 Hz and 6.5 − 7.5 Hz, respectively) were lower in the Wisket animals compared to controls.

The separate analysis of the theta band by substages revealed significant effects of group [*F*_(1_,_896)_ = 123.00; *p* < 0.001], frequency [*F*_(31_,_896)_ = 63.92; *p* < 0.001], substage [*F*_(1_,_896)_ = 120.98; *p* < 0.001], group and frequency interaction [*F*_(31_,_896)_ = 5.71; *p* < 0.001], group and substage interaction [*F*_(1_,_896)_ = 9.44; *p* < 0.005] and frequency and substage interaction [*F*_(31_,_896)_ = 3.64; *p* < 0.001]. Thus, the Wisket animals in both active and quiet substages had significantly lower total power (6.25 − 7.75 and 6.62 − 7.38 Hz, respectively, Figure 1F).

**FIGURE 2:**
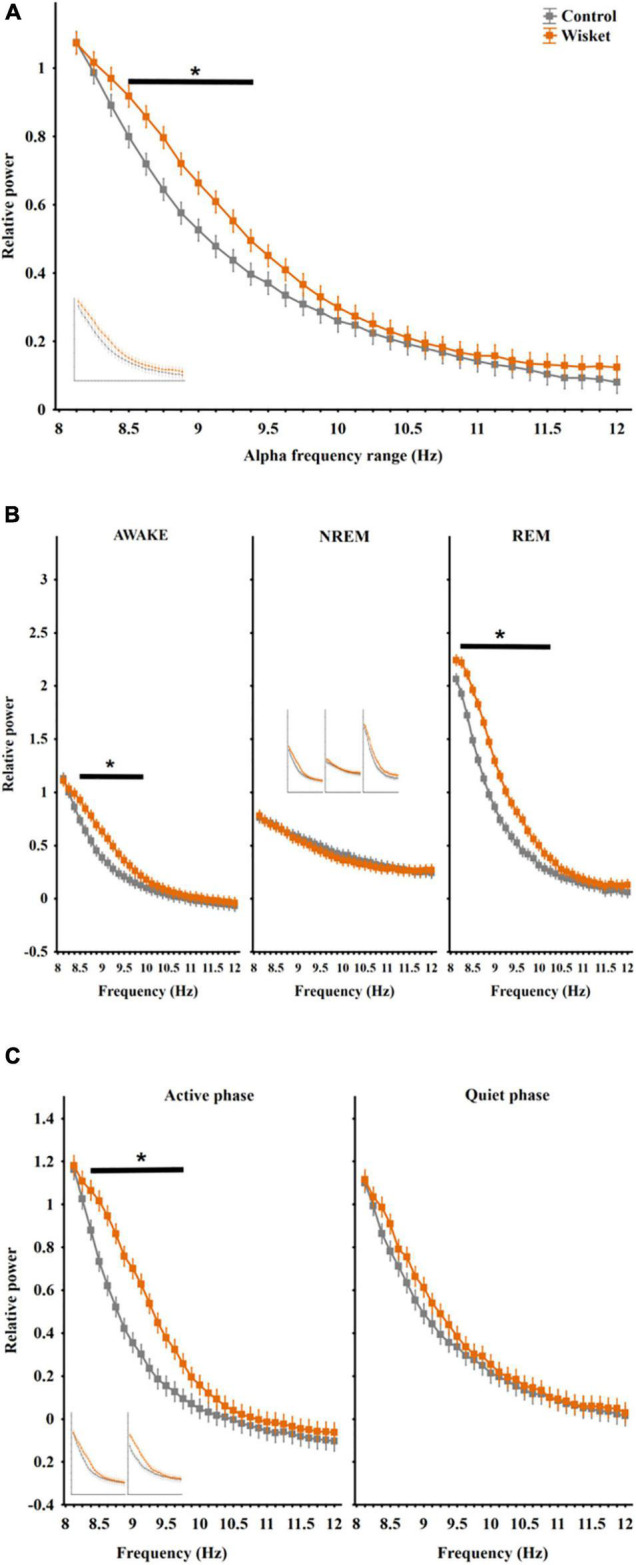
Relative EEG power differences between the two groups at the alpha frequency band. **(A)** Total relative alpha power (8–12 Hz). **(B)** Alpha power in different stages panel. **(C)** Alpha power in active and quiet awake substages. Curves inserted in reduced size show the results obtained from unmatched control and Wisket animals. The symbol * shows significant (*p*° < °0.05) differences between the two groups.

Regarding the total relative power in the alpha band (8 − 12 Hz), factorial ANOVA showed significant effects of group [*F*_(1_,_448)_ = 52.00; *p* < 0.001] and frequency [*F*_(31_,_448)_ = 170.77; *p* < 0.001]. The Wisket animals had significantly higher relative power compared to control ones at the lower range of alpha band (8.5 − 9.38 Hz interval; Figure 2A).

The analysis of the alpha power by stages revealed significant effects of group [*F*_(1_,_1344)_ = 167.26; *p* < 0.001], frequency [*F*_(31_,_1344)_ = 354.09; *p* < 0.001], stage [*F*_(2_,_1344)_ = 980.07; *p* < 0.001], group and frequency interaction [*F*_(31_,_1344)_ = 3.94; *p* < 0.001], group and stage interaction [*F*_(2_,_1344)_ = 81.04; *p* < 0.001], frequency and stage interaction [*F*_(62_,_1344)_ = 48.09; *p* < 0.001] and group, frequency, and stage interaction [*F*_(62_,_1344)_ = 1.63; *p* < 0.005]. The pattern of the alpha spectrum differed between the stages with the lowest rise of the curve during NREM, and the Wisket animals had higher relative alpha power in awake and REM stages in the lower frequency bands (8.38 − 9.75 Hz and 8.25 − 10 Hz, respectively) compared to control rats (Figure 2B).

**FIGURE 3:**
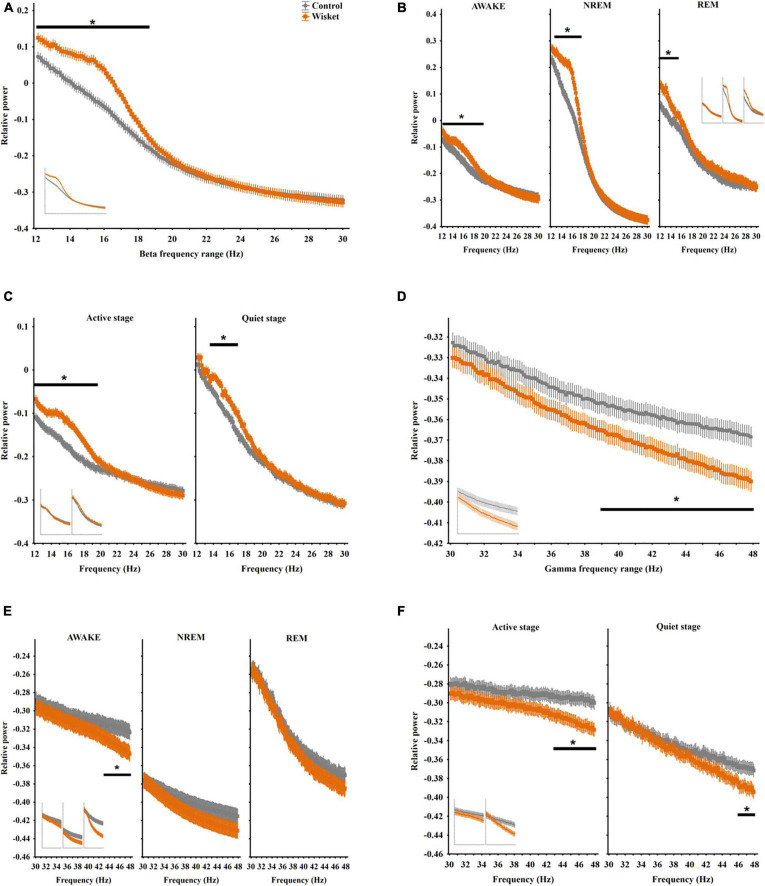
Relative EEG power differences between the two groups at the beta and gamma frequency bands. **(A)** Total relative beta power (12–30 Hz). **(B)** Beta power in different stages. **(C)** Beta power in active and quiet awake substages. **(D)** Total relative gamma power (30–48 Hz). **(E)** Gamma power in different stages. **(F)** Gamma power in active and quiet awake substages. Curves inserted in reduced size show the results obtained from unmatched control and Wisket animals. The symbol * shows significant (*p*° < °0.05) differences between the two groups.

The separate analysis of the alpha band by substages disclosed significant effects of group [*F*_(1_,_896)_ = 126.58; *p* < 0.001], frequency [*F*_(31_,_896)_ = 230.56; *p* < 0.001), substage [*F*_(1_,_896)_ = 92.76; *p* < 0.001], group and frequency interaction [*F*_(31_,_896)_ = 2.90; *p* < 0.001], group and substage interaction [*F*_(1_,_896)_ = 30.94; *p* < 0.001], and frequency and substage interaction [*F*_(31_,_896)_ = 2.02; *p* < 0.001]. The *post hoc* comparison revealed that the Wisket animals had significantly higher relative power at the lower frequency alpha band (8.38 − 9.75 Hz) compared to controls during the active awake substage (Figure 2C). Regarding the relative power during the whole investigated period in the beta band (12–30 Hz), factorial ANOVA showed significant effects of group [*F*_(1_,_2016)_ = 344.18; *p* < 0.001], frequency [*F*_(143_,_2016)_ = 235.91; *p* < 0.001], and group and frequency interaction [*F*_(143_,_2016)_ = 4.18; *p* < 0.001]. Thus, the Wisket animals had significantly higher total relative power compared to control ones at the lower frequency beta band (12.12 − 18.38 Hz interval; Figure 3A).

The analysis of the beta power by stages showed significant effects of group [*F*_(1_,_6048)_ = 625.89; *p* < 0.001], frequency [*F*_(143_,_6048)_ = 390.36; *p* < 0.001], stage [*F*_(2_,_6048)_ = 1415.83; *p* < 0.001), group and frequency interaction [*F*_(143_,_6048)_ = 4.24; *p* < 0.001], group and stage interaction [*F*_(2_,_6048)_ = 35.80; *p* < 0.001], and frequency and stage interaction [*F*_(286_,_6048)_ = 44.05; *p* < 0.001]. The pattern of the beta spectrum differed between the stages with the highest steepness of the curve during NREM, and the Wisket animals had higher relative power in all stages at the lower beta frequency band (awake: 12.12 − 19.38 Hz, NREM: 12.88 − 17.5 Hz, REM: 12.12 − 15.75 Hz; Figure 3B).

The separate analysis of the beta band by substages revealed significant effects of group [*F*_(1_,_4032)_ = 541.50; *p* < 0.001], frequency [*F*_(143_,_4032)_ = 312.70; *p* < 0.001], substage [*F*_(1_,_4032)_ = 649.00; *p* < 0.001], group and frequency interaction [*F*_(143_,_4032)_ = 5.00; *p* < 0.001], and frequency and substage interaction [*F*_(143_,_4032)_ = 20.90; *p* < 0.001]. Thus, the Wisket animals had significantly higher relative power at the lower frequency beta band compared to controls primarily during the active awake substage (active: 12.12 − 19.38 Hz, quiet: 12.38 − 12.5 Hz; Figure 3C).

As regards the relative power during the whole period in the gamma band (30 − 48 Hz), factorial ANOVA showed significant effects of group [*F*_(1_,_2002)_ = 588.00; *p* < 0.001) and frequency [*F*_(142_,_2002)_ = 20.00; *p* < 0.001). The *post hoc* analysis disclosed that the Wisket animals had significantly lower whole relative power compared to control ones at the higher gamma frequency band (39 − 48 Hz interval; Figure 3D).

The analysis of the gamma power by stages revealed significant effects of group [*F*_(1_,_6006)_ = 332.00; *p* < 0.001], frequency [*F*_(142_,_6006)_ = 39.00; *p* < 0.001], stage [*F*_(2_,_6006)_ = 9538.00; *p* < 0.001], and frequency and stage interaction [*F*_(284_,_6006)_ = 7.00; *p* < 0.001]. The *post hoc* analysis showed that the pattern of the gamma spectrum differed between the stages with the lowest steepness of curve values during NREM, and the Wisket animals had lower relative gamma power, primarily during the awake stage at the higher frequency range (44.25 − 48 Hz; Figure 3E).

The separate analysis of the gamma band by substages revealed significant effects of group [*F*_(1_,_4004)_ = 478.00; *p* < 0.001], frequency [*F*_(142_,_4004)_ = 17.00; *p* < 0.001], substage [*F*_(1_,_4004)_ = 8230.00; *p* < 0.001], group and substage interaction [*F*_(1_,_4004)_ = 54.00; *p* < 0.001], and frequency and substage interaction [*F*_(142_,_4004)_ = 3.00; *p* < 0.001]. Thus, the Wisket animals had significantly lower relative power at the higher frequency gamma band compared to controls, primarily during the active awake substage (active: 42.62 − 48 Hz, quiet: 46.16 − 48 Hz; Figure 3F).

Regarding the data of unmatched series, the curves were inserted into the figures in reduced size (Figures 1–3). Most of the observed changes in the matched Wisket animals could also be obtained in the unmatched animals.

## Discussion

The main objective of the present study was to assess changes in sleep architecture and oscillatory pattern of a multiple hit schizophrenia rat model. The Wisket animals have a clear light-dark cycle similar to controls, and their wake-sleep rhythm showed only a tendency to alterations. However, important signs of abnormalities in their oscillatory pattern were obtained by applying a fine-grain analysis within different frequency bands. In spite of the lack of the diurnal variation in the oscillation in both groups, the Wisket rats showed higher relative power in the low-frequency delta, alpha, and beta bands and lower relative power in the high-frequency theta and gamma bands in most stages and substages. The same pattern of differences could be observed between the unmatched groups even with the low number of animals, suggesting that these alterations are stabile with high reproducibility. Furthermore, the automated categorization of the stages and substages had a high level of correlation with the manual classification.

The automated sleep stage discrimination was carried out using software developed in-house, to assign each epoch to one of the three stages. While visual EEG classification is still considered the gold standard for sleep scoring, the considerable progress in computational technologies has made possible the improvement of automated sleep scoring algorithms (Miladinović et al., 2019; Yamabe et al., 2019). Conventional methods for identifying and removing EEG artifacts are subjective and time consuming, that are commonly dealt with based on qualitative and subjective criteria. The artifacts can be repaired or rejected (Jas et al., 2017). While the method of excluding artifacts from the analysis is very often employed, it leads to the loss of several data. Therefore, we presented an automated algorithm for unified reception and repairing these artifacts in EEG signals. The algorithm allowed us to automate the preprocessing of EEG data. The automated nature of this method minimized the burden of human inspection, supporting scalability and reliability data analysis.

It is very important for an automatic classification to achieve the accuracy level required for practical research usage. Generally, the inter-rater agreement rate of the manual sleep stage scoring results in rodents is reported to be approximately 95%. While we found 92.8% agreement between the manual and automated classification, we assume that this level should also provide reliable data. This was supported by the very similar data obtained with different groups of animals.

Normal wake-sleep rhythm is an important physiological process underlying the maintenance of physical, mental, and emotional health. Sleep disorder is one of the most common signs in patients with schizophrenia, that correlates with cognitive and affective abnormalities (Cohrs, 2008; Poulin et al., 2010; Pritchett et al., 2012; Winsky-Sommerer et al., 2019). Most of the studies suggest reduced sleep duration and enhanced sleep fragmentation; however, sleep-onset insomnia can counterintuitively increase total sleeping time in some patients compared to healthy controls (Poulin et al., 2010). Although direct comparison of clinical and preclinical data is difficult, especially because rats, unlike humans, are polycyclic sleepers, the Wisket animals had almost normal diurnal variation in their sleep-wake pattern. This is in agreement with some previous reports obtained in different schizophrenia models, including DISC1 gene mutant mice and rats with prenatal intervention or neonatal hippocampal lesion (Ahnaou et al., 2007; Phillips et al., 2012b; Valdés-Cruz et al., 2012; Profitt et al., 2016; Dittrich et al., 2017). The Wisket rats spent a slightly reduced time awake during the light phase accompanied by a moderately prolonged period in NREM sleep. Furthermore, a tendency of decreased REM sleep duration was detected compared to the control group. This phenomenon was accompanied by a mild decrease in the number of episodes in all stages. Apparently, these results seem to be in contrast with some human data or results obtained in animal models of schizophrenia (Phillips et al., 2012a; Pritchett et al., 2012; Profitt et al., 2016; Winsky-Sommerer et al., 2019). However, the decreased incidence of REM sleep episodes accompanied by enhanced NREM duration was also detected in mGLUR5 mutant mice (Ahnaou et al., 2015b; Ang et al., 2018). These signs may be derived from a chronic imbalance between the ascending and descending systems, of which activities are known to trigger or to dampen the occurrence of NREM and REM sleep states, respectively (Brown et al., 2012). Therefore, less sequential transitions in the NREM-REM sleep cycle might correlate with impaired cognition processes obtained in Wisket animals (Kekesi et al., 2015; Horvath et al., 2017). The unchanged fragmentation of the episodes seems to be in contrast with the earlier results obtained with telemetry, where decreased motor activity with the fragmented pattern was observed in the Wisket animals (Horvath et al., 2015). However, telemetry is only suited to monitor gross motor activity, it cannot differentiate between the activity stages, and it cannot detect fine movements (Horvath et al., 2015). Furthermore, the micro-arousals (shorter than 8 s long fragments of the awake stage) that were not involved in the EEG analysis might have modified the results (Kang et al., 2015).

In summary, the wake-sleep phenotype of Wisket rats may mimic only a slight abnormality observed in a small subset of patients with schizophrenia but does not reflect disease-related sleep abnormalities overall (Kaskie et al., 2017). However, all these changes might have a significant influence on the oscillatory pattern in different frequency bands. Neuronal oscillations represent a fundamental mechanism enabling coordinated activity during normal brain functioning and are, therefore, an instrumental research target for neuronal and neuropsychiatric disorders. The oscillatory pattern may depend on the strength and kinetics of inhibitory and excitatory synaptic interactions and data suggest that ineffective inhibitory control of sensory processing is characteristic in schizophrenia (Gyorgy and Andreas, 2004; Orekhova et al., 2008; Uhlhaas and Singer, 2013). Altered oscillatory activity in the different EEG frequency bands has been reported in patients, which may contribute to their abnormal sensory and cognitive performance (Felder et al., 1994; Sponheim et al., 1994, 2003; Omori et al., 1995; Symond et al., 2005; Rockstroh et al., 2007; Bob et al., 2008; Boutros et al., 2008; Lakatos et al., 2013; Uhlhaas and Singer, 2013; Csukly et al., 2016). Unfortunately, the results are controversial and they may depend on the subtypes or phases of this disease (Adler et al., 1982; Braff and Geyer, 1990; Sponheim et al., 2003; Başar, 2013; Uhlhaas and Singer, 2013). In agreement with earlier studies, the oscillatory pattern did not show differences between the dark and light phases neither in the control nor in the Wisket groups (Ahnaou et al., 2015a; Profitt et al., 2016; Aguilar et al., 2020). However, several differences in EEG power spectra across the three different stages were detected between the two groups but at different frequency ranges. Oscillations in different frequency bands may have different underlying mechanisms, which subserve different functions. Thus, the differences in cortical oscillations might suggest very complex disturbances in the Wisket animals.

Regarding the delta wave, it is originated from the reticular nucleus of the thalamus, where GABAergic neurons are tonically activated *via* NMDA receptors, thereby regulating the activity of thalamic relay neurons projecting to the prefrontal cortex (Lisman, 2012). Thus, the interplay among spontaneous synaptic inputs, intrinsic neural properties, and coupled thalamocortical network oscillations generates EEG power in this frequency range (Crunelli and Hughes, 2010). It is mainly associated with brain quiescence and the closing of thalamic gates for external input, but such activity has also been related to unexpectedly high levels of spontaneous neuronal activity, which may serve important cognitive processes such as memory consolidation (Steriade, 2006). This frequency band during the NREM stage plays important role in the recovery function of sleep and the beneficial cognitive effects of sleep (Borbély, 2001; Borbély et al., 2016).

Augmented low-frequency oscillations were observed in unmedicated, first-episode, and chronic patients with schizophrenia (Sponheim et al., 1994; Knott et al., 2001; Rockstroh et al., 2007; Boutros et al., 2008; Newson and Thiagarajan, 2019). Other data suggest that delta band power increased in patient with negative symptoms, while it decreased in patients with positive symptom domains (Begic et al., 2000). It was suggested that the abnormal slow wave activity is a manifestation of disrupted formation of neural circuits, facilitating histological anomalies, like gray matter loss.

In our experiments, only a tendency of enhancement in the delta band power was found during the awake stage (at both the active and quiet substages) compared to the controls. However, the Wisket animals showed significantly enhanced delta activity in its lower frequency range during the NREM phase, while it decreased in the higher frequency range of the delta band during the NREM and REM stages. The higher power in the lower frequency delta band might suggest enhanced requirements of sleep (Borbély, 2001). The reduced delta power during NREM sleep at the higher frequency range could arise from cortical dysfunction, altered thalamocortical input, or both, while the enhancement in the slow oscillations at the higher delta band was thought to indicate less exchange of excitation (Rockstroh et al., 2007). Regarding the earlier preclinical studies, a tendency of enhancement in delta power in both wakefulness and NREM sleep was found 2 months after neonatal hippocampal lesion (Ahnaou et al., 2007). STOP mutant mice also showed an increase in relative power in the delta band (0.5–4 Hz) during NREM (Profitt et al., 2016). In agreement with our data, AMPA receptor mutant mice had higher power in a lower frequency range (0.75–1.5 Hz), while mGLUR5 receptor mutant mice had lower delta power at a higher frequency range during NREM compared to control animals (Ahnaou et al., 2015b; Ang et al., 2018).

The cortico-hippocampal circuits are key generators of theta rhythm, which has an important role in cognition and memory formation (Buzsáki, 2002; Gyorgy and Andreas, 2004; Buzsáki and Moser, 2013). Theta oscillations dominate during motor activity or when the rats remain motionless but alert (Buzsáki, 2002). REM sleep in rodents is characterized by a prominent theta rhythm in EEG recording (as was observed in both groups of rats), reflecting hippocampal activity (Boyce et al., 2016). Its generation depends on GABA neurons in the medial septum projecting to the hippocampus, thus, the silencing of these neurons during REM sleep led to decreased theta power and impairments in memory consolidation.

Inconsistent changes in theta band power were observed in patients with schizophrenia (Winterer and Weinberger, 2004; Javitt et al., 2008; Lakatos et al., 2013; Grove et al., 2021) depending on the dominant symptom domain, i.e., in patients with positive symptoms, there was no change, while in case of negative signs, it increased (Gerez and Tello, 1995; Begic et al., 2000; Winterer and Weinberger, 2004; Javitt et al., 2008; Lakatos et al., 2013; Grove et al., 2021).

In Wisket rats, the significantly lower relative power of the higher frequency band was observed during the awake (both the active and quiet substages) and REM stages. In contrast, they had significantly lower power at the lower frequency range of theta band during NREM. Optogenetic inhibition of REM theta power impairs sleep-dependent memory consolidation in mice (Boyce et al., 2016). Therefore, our observation of decreased theta power in REM might explain the memory impairment in Wisket rats reported earlier (Kekesi et al., 2015; Horvath et al., 2017). Regarding the schizophrenia models, the results are controversial. Enhancement of power in theta bands was observed in both wakefulness and NREM sleep in neonatal hippocampal lesioned rats (Ahnaou et al., 2007). In contrast, but agreement with our data, STOP or AMPA receptor mutant mice had lower relative power in the theta band (Profitt et al., 2016; Ang et al., 2018), suggesting reduced arousal level during the active stage (Leemburg et al., 2010).

The oscillation in the alpha band is related primarily to the thalamus, thus, the alterations in this frequency band may suggest dysfunction of the inhibitory input of thalamic neurons (Markand, 1990; Begic et al., 2000). There are limited and inconsistent data regarding the role and significance of alpha oscillations in schizophrenia. Either higher alpha power associated with negative symptoms or reduced alpha band activity in patients with both negative or positive signs of schizophrenia were reported (Gerez and Tello, 1995; Begic et al., 2000; Boutros et al., 2008; Featherstone et al., 2015; Newson and Thiagarajan, 2019).

While Wisket animals showed significantly enhanced relative power at the lower frequency alpha band during awake (primarily in active substage) and REM stages, it might be related to the negative symptoms. In agreement with our results, hippocampal lesion increased the alpha power during the awake stage (Ahnaou et al., 2007), but AMPA receptor or STOP mutant schizophrenia model mice demonstrated decreases in the alpha power during the REM phase (Profitt et al., 2016; Ang et al., 2018).

Beta oscillations are believed to be generated broadly across multiple neocortical structures and are involved in adaptation to repetitive sensory stimuli, attention, affective processing, alertness, and synchronization of large ensembles of neurons (Uhlhaas and Singer, 2013; Featherstone et al., 2015). Both decreased and increased beta oscillatory activities were detected in patients with schizophrenia (Javitt et al., 2008; Uhlhaas and Singer, 2013; Csukly et al., 2016). The enhancement in the beta band might be due to global cortical hyperexcitability or attention disturbances observed in these patients (Begic et al., 2000; Newson and Thiagarajan, 2019).

Our data clearly showed that Wisket animals had significantly enhanced beta power at the lower frequency range in all stages compared to control animals, but this difference was most prominent during active awake substage. These alterations might play role in the impaired cognitive behavior of Wisket model rats observed earlier (Horvath et al., 2019; Saravanapandian et al., 2020). Preclinical data are also inconsistent in the beta wave changes. STOP and AMPA receptor and voltage-gated calcium channel mutant mice had lower beta power during NREM and/or REM stages, the AMPA receptor mutant animals had higher beta power during awake, while the calcium channel mutant mice had lower power in this stage (Kumar et al., 2015; Profitt et al., 2016; Ang et al., 2018). In contrast, the hippocampal lesion did not cause changes in this frequency band (Ahnaou et al., 2007).

Gamma oscillations have received great interest because of their role in sensory and cognitive functions such as selective attention, short- and long-term memory, and multisensory integration (Traub et al., 1999; Gyorgy and Andreas, 2004; Uhlhaas and Singer, 2013; Featherstone et al., 2015; Tatard-Leitman et al., 2015). The primary generators of gamma oscillations are in the cortex, where subsets of GABAergic interneurons modulate the glutamatergic pyramidal cell activity, while the pyramidal neurons appear to control the strength, duration, and long-range synchronization of the GABAergic interneurons. Abnormal gamma oscillations have been associated with positive, negative, and cognitive symptoms of schizophrenia (Lee et al., 2001; Uhlhaas and Singer, 2013; Featherstone et al., 2015). Many studies observed reduced oscillatory activity that may reflect the deficits in cognitive and sensory processing related to negative symptoms in schizophrenia (Kwon et al., 1999; Symond et al., 2005; Hong et al., 2010; Koychev et al., 2012; Uhlhaas and Singer, 2013). However, contrasting findings of increased gamma activities in schizophrenia also exist, and it is reportedly relevant to positive symptoms (Gerez and Tello, 1995; Norra et al., 2004; Javitt et al., 2008; Başar, 2013; Lakatos et al., 2013; Baradits et al., 2019).

Our results showed that Wisket animals had a significant decrease in the relative power of the higher band of gamma wave (between 39 and 48 Hz) compared to the controls, which was dominant during the awake stage (primarily in the active substage). It is well-known that gamma band oscillations are associated with multiple cognitive functions, and gamma activity increases during alert or attentive wakefulness, especially above 40 Hz (Maloney et al., 1997; Uhlhaas and Singer, 2013). Therefore, this phenomenon in Wisket rats may indicate a lower level of arousal or alertness during wakefulness, but could also be related to the impaired cognitive functions obtained earlier in the Wisket animals (Franken et al., 1994; Horvath et al., 2019). Earlier preclinical data are controverting in this respect. In agreement with our data, the animals after chronic NMDA antagonists treatment or with voltage-gated calcium channel mutation also displayed no change or reduction in gamma power (Kittelberger et al., 2012; Featherstone et al., 2014; Kumar et al., 2015; Sullivan et al., 2015), but elevated gamma activity was recorded during sleep in mGluR5 mutant mice (Aguilar et al., 2020).

## Limitations

There are several limitations in our recording system: the maximum recording duration and the limited number of channels, and a lack of deep hippocampal electrodes. Furthermore, as we used a tethered option rather than wireless recording, the rats were singly housed in cages, to provide appropriate circumstances for fully recovering after the surgery and prevent the destruction of the EEG devices and cables. It is well known that long-term single housing (more than 1 week), especially in young animals, affects several biological parameters (Weiss et al., 2004; Makinodan et al., 2012; Yamamuro et al., 2018). However, in this study, the social isolation was performed during adulthood for only 1 week, and the animals were kept in visual, auditory, and olfactory contact with other rats during the whole experimental period to minimalize the effect of isolation rearing (Hurst et al., 1999). It should also be mentioned that we used a limited gamma frequency range in our analysis; therefore, it could not be excluded from further impairments in the higher frequency band in Wisket animals. A further limitation is the lower sampling rate of the EEG recordings (128 Hz), which would have limited the fine analyses of the oscillatory pattern in the higher gamma frequency band.

## Conclusion

Our data first characterized the sleep-wake rhythm and oscillatory pattern of a multiple hit rat model of schizophrenia. The results indicate that the Wisket rats have only a tendency to alterations in their sleep-wake pattern with normal circadian rhythm. On the other hand, the investigation of the oscillatory patterns in different frequency bands revealed complex alterations in all stages and substages of the sleep-wake rhythm.

Wakefulness and sleep are promoted by the ascending arousal and descending inhibitory pathways, respectively, operating with several neurotransmitters, including dopamine, glutamate, and GABA systems (Saper et al., 2005). The Wisket rats have impairment in several receptor systems (Szûcs et al., 2016a,^2020^; Büki et al., 2019; Banki et al., 2020; Horvath et al., 2021b), which might be involved in both the development of schizophrenia-like behavioral signs and the connected particular brain oscillation (Svensson et al., 1987; Hajos et al., 2008; Kittelberger et al., 2012).

A better understanding of the characteristics of sleep disturbance and oscillatory activity in this schizophrenia model may help to address fundamental relationships among the behavioral, neurochemical, and electrophysiological parameters, as well as to develop novel drug targets and therapies to treat this disorder.

## Data Availability Statement

The raw data supporting the conclusions of this article will be made available by the authors, without undue reservation.

## Ethics Statement

The animal study was reviewed and approved by Hungarian Ethical Committee for Animal Research.

## Author Contributions

LA contributed to data collection, interpretation of the results, and manual classification. MC-N developed data classification software and tested by comparison with manual classification. BB did animal surgery, conducted the experiments, and developed the data recording system. GyK developed data classification software and contributed to statistical analysis. LN supervised software development and performed proofreading. AN supervised the experiments and manual classification of data and performed proofreading. GaK contributed to the interpretation of the results and performed proofreading. AB contributed to statistical analysis. GH conceived of the presented idea, planned the experiments, performed statistical analysis, and wrote the first draft of the manuscript.

## Conflict of Interest

The authors declare that the research was conducted in the absence of any commercial or financial relationships that could be construed as a potential conflict of interest.

## Publisher’s Note

All claims expressed in this article are solely those of the authors and do not necessarily represent those of their affiliated organizations, or those of the publisher, the editors and the reviewers. Any product that may be evaluated in this article, or claim that may be made by its manufacturer, is not guaranteed or endorsed by the publisher.

## Supplementary Material

Supplementary Figure 1 Interventions in the Wisket animals to develop the triple-hit schizophrenia model. TF, tail-flick test to determine pain sensitivity; PPI, prepulse inhibition test to measure sensory gating; Ambitus, a reward-based cognitive test to determine motor activity and learning capability.
