# Supplementary figures and images for: Sleep-Wake Rhythm and Oscillatory Pattern Analysis in a Multiple Hit Schizophrenia Rat Model (Wisket)
Source: Front Behav Neurosci. 2022 Jan 28;15:799271. doi: 10.3389/fnbeh.2021.799271 (PMC8831724; doi:10.3389/fnbeh.2021.799271)

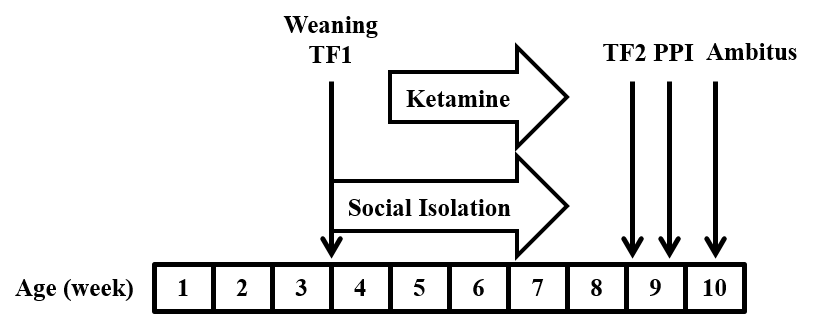

Supplement: Supplementary Figure 1 — Interventions in the Wisket animals to develop the triple-hit schizophrenia model. TF, tail-flick test to determine pain sensitivity; PPI, prepulse inhibition test to measure sensory gating; Ambitus, a reward-based cognitive test to determine motor activity and learning capability. [file Image_1.TIF]
